# Neural Responses of Pet Dogs Witnessing Their Caregiver’s Positive Interactions with a Conspecific: An fMRI Study

**DOI:** 10.1093/texcom/tgab047

**Published:** 2021-07-19

**Authors:** Sabrina Karl, Ronald Sladky, Claus Lamm, Ludwig Huber

**Affiliations:** Comparative Cognition, Messerli Research Institute, University of Veterinary Medicine Vienna, Medical University of Vienna, University of Vienna, 1210 Vienna, Austria; Social, Cognitive and Affective Neuroscience Unit, Department of Cognition, Emotion, and Methods in Psychology, Faculty of Psychology, University of Vienna, 1010 Vienna, Austria; Social, Cognitive and Affective Neuroscience Unit, Department of Cognition, Emotion, and Methods in Psychology, Faculty of Psychology, University of Vienna, 1010 Vienna, Austria; Comparative Cognition, Messerli Research Institute, University of Veterinary Medicine Vienna, Medical University of Vienna, University of Vienna, 1210 Vienna, Austria

**Keywords:** attachment, canine neuroimaging, human–animal interaction

## Abstract

We have limited knowledge on how dogs perceive humans and their actions. Various researchers investigated how they process human facial expressions, but their brain responses to complex social scenarios remain unclear. While undergoing fMRI, we exposed pet dogs to videos showing positive social and neutral nonsocial interactions between their caregivers and another conspecific. Our main interest was how the dogs responded to their caregivers (compared to a stranger) engaging in a pleasant interaction with another dog that could be seen as social rival. We hypothesized that the dogs would show activation increases in limbic areas such as the amygdala, hypothalamus, and insula and likely show higher attention and arousal during the positive caregiver–dog interaction. When contrasting the social with the nonsocial interaction, we found increased activations in the left amygdala and the insular cortex. Crucially, the dogs’ hypothalamus showed strongest activation when the caregiver engaged in a positive social interaction. These findings indicate that dogs are sensitive to social affective human–dog interactions and likely show higher valence attribution and arousal in a situation possibly perceived as a potential threat to their caregiver bonds. Our study provides a first window into the neural correlates of social and emotional processing in dogs.

## Introduction

Given the particular features of the anthropogenic habitat of companion dogs, proper communication with humans is of crucial importance. In recent years, researchers have therefore become increasingly interested in how dogs understand humans, especially as they show remarkable abilities for interacting and communicating with us. The main focus of previous research was on how dogs perceive elements of their environment, learn about it, and use this knowledge to make informed decisions about proper behavior (for reviews, see Bensky et al. 2013; Huber 2016; Lea and Osthaus 2018; Marshall-Pescini and Kaminski 2014). For proper communication between a dog and its human partner, the dog needs to detect predictable changes in the behavior of its counterpart in response to certain signals from her/him (e.g., Virányi et al. 2004; Udell and Wynne 2008; Dorey et al. 2010; Kaminski et al. 2012).

Perhaps the most extensively studied topic in this regard is how dogs perceive the human face and what kind of information they extract from this multifaceted signal to understand who we are (identity; Huber et al. 2013; Mongillo et al. 2017; Eatherington et al. 2020), whether we are attentive toward them or not (e.g., Schwab and Huber 2006; Kaminski and Nitzschner 2013), or how they assess in what parts of the environment we are interested in depending on our looking behavior (gaze following; e.g., Wallis et al. 2015) or our emotional reaction (social referencing; e.g., Merola et al. 2012a; Merola et al. 2012b) and what humans have seen and therefore know (perspective taking; Catala et al. 2017; Maginnity and Grace 2014). Recent research has revealed that dogs cannot only discriminate human emotional facial expressions (Müller et al. 2015), but that they can also rely on human facial expressions when making decisions about approaching other objects (Merola et al. 2012a). A few studies have even suggested that dogs recognize or understand the emotional content and meaning of human faces (Albuquerque et al. 2016; Barber et al. 2016; Somppi et al. 2016). Though future research will have to confirm this, it seems safe to argue that all these kinds of information are useful and beneficial for proper communication and the development of a positive human–dog relationship.

The relationship between the dog and its human partner has proven to be important in a variety of situations (Range et al. 2007; Horn et al. 2013), especially in those that require high attention toward humans, such as in learning from them (Kubinyi et al. 2003; Tópal et al. 2006; Huber et al. 2009; Range et al. 2011; Fugazza and Miklósi 2014; Huber et al. 2014) and in cooperation with humans (Naderi et al. 2001; Bräuer et al. 2013; Ostojić and Clayton 2014). The influence of the familiarity or the relationship with the human demonstrator has recently been highlighted in tests of overimitation, in which dogs copy unnecessary or causally irrelevant actions more if the human demonstrator is familiar to them than if not (Huber et al. 2018; Huber et al. 2020).

In general, it has been hypothesized that companion dogs may form a special relationship with their human caregivers that bears a remarkable resemblance to the attachment bond of human infants with their mothers (Bowlby 1958), because they are not only dependent on human care, but their behavior is specifically geared to engage their human partner’s caregiving system (Topál et al. 1998; Prato-Previde et al. 2003; Horn et al. 2013; Prato-Previde and Valsecchi 2014). However, until very recently, this human–dog attachment hypothesis has only been based on behavioral and endocrinal evidence (for review, see Prato-Previde and Valsecchi 2014).

Very recently, Karl et al. (2020) published the results of a multimethod approach combining neuroimaging (fMRI), eye-tracking, and behavioral preference tests to explore the engagement of an attachment-like system in dogs when seeing human faces. Rather than presenting static images, the authors showed morphing videos of the caregiver, a familiar person, and a stranger while they changed their emotional facial expression from neutral to either happy or angry. The fMRI experiment revealed that, regardless of emotion, the viewing of the caregiver led to activations of brain regions associated with emotion and attachment processing in humans. In contrast, the stranger elicited activation mainly in brain regions related to visual and motor processing, and the familiar person relatively weak activations overall.

With the present study, we aimed at extending these findings about an attachment-like system in dogs by having pet dogs watch video clips showing their caregiver while she or he engaged in a positive social or in a neutral nonsocial interaction with another dog. Video clips seemed to us much better than static images due to their realistic content and the fact that dogs are much more attentive to dynamic than static stimuli (Völter et al. 2020). We compared brain responses to such videos to matched control conditions, which showed an unknown person (stranger) during the same types of interactions with the same dog. Note that in this setup, the emotional content of the videos and the interactions was not only provided by the human’s facial expression (as in our previous study) but also by her/his behavior in the interaction with another dog. This study design has originated from Bowlby’s observation that, “in most young children the mere sight of mother holding another baby in her arms is enough to elicit strong attachment behavior” (Bowlby 1969, p. 215). Therefore, we were interested in two main aspects. Firstly, we examined the effect of the human’s identity, that is, whether the dog’s caregiver or a stranger is shown to interact with another dog. Secondly, we were interested in the dog’s perception and the putative (emotional) interpretation of the human–dog interaction, which either consisted of petting the other dog as an example for a positive social interaction, or a neutral nonsocial and nonaffective interaction that resembled a brief clinical examination (“veterinary check”) of the other dog. Note, nonsocial is meant in the sense that the intent of this interaction was not to establish a social–affective bond. Altogether, we hypothesized to find activation patterns that could be interpreted as a response to the specific donor–receiver–behavior combination. Specifically, we hypothesized that witnessing the attachment figure enjoying a pleasant interaction with another dog would result in increased attention and alertness, and negative affective responses of the perceiver dog. This hypothesis was derived from the assumption that in this setting, the other dog is perceived as a social rival for the attention and affection of their main attachment figure. In humans, the latter would trigger negative effects, as individuals might react with expressions of sadness, fear, or anger and probably overt behaviors to regain the attachment figure’s affection, exclusive attention, and allegiance (Hupka 1984; Hart 2010; Mize et al. 2014). Therefore, the perceiver dogs were expected to react in negative ways and with increased attention and arousal, and that this could be related to (precursors of) anger or fear of losing an important social bond (Parrott and Smith 1993; Mize and Jones 2012; Hart 2016a).

That dogs may show negative emotions to the interactions between humans and other dogs has been shown in tests of inequity aversion, the resistance to inequitable outcomes (Range et al. 2009). Dogs evidenced signs of distress such as scratching themselves, yawning, and licking the mouth in situations in which the other dog still received food reward for giving the paw when they themselves were not rewarded anymore for the same action. Control conditions demonstrated that the subjects reacted to the inequity rather than to the sudden failure of reward. It has been assumed that dogs when exhibiting inequity aversion are not necessarily keeping track of past interactions but rather behave as a consequence of the positive or negative emotions induced by such situations (McGetrick et al. 2020). We therefore hypothesized that the subjects in our study would also show differential neural activities indicative of high arousal and negative affectivity, especially higher neural activity in brain regions such as the amygdala, the hypothalamus, and the insular cortex, areas associated with affective salience and evaluation, interoceptive processes, and autonomic regulation in humans (Rilling et al. 2004; Takahashi et al. 2006; Marazziti et al. 2013; Saper and Lowell 2014; Janak and Tye 2015; Maninger et al. 2017; Zheng et al. 2019).

Recent studies have pursued related research questions by showing various human–dog interactions with either a fake or a real dog to pet dogs to investigate their mainly behavioral responses (Harris and Prouvost 2014; Abdai et al. 2018; Prato-Previde, Nicotra, Fusar Poli, et al. 2018a; Prato-Previde, Nicotra, Pelosi, et al. 2018b; Bastos et al. 2021). In some of these studies, the subjects displayed behavioral responses such as intervening and attempts to get between the interacting parties (Harris and Prouvost 2014; Abdai et al. 2018), which may indicate higher alertness and arousal of the dogs. These dog behaviors were shown in the presence of the dogs’ caregivers and were especially more frequent in the social interaction conditions compared with the nonsocial interactions, e.g., with objects. Hence, they have been interpreted as “jealousy”- or protective-like behaviors as seen in human infants in similar situations to regain attention and to protect the valued relationship and the social bond with the mother (for review, see Hart 2016b).

A recent dog fMRI study found increased amygdala activity of dogs observing their caregiver giving treats to a fake dog in comparison to putting treats into a bucket (Cook et al. 2018). However, this effect was restricted to dogs that showed a high dog–dog aggression score (Canine Behavioral Assessment & Research Questionnaire—C-BARQ; Hsu and Serpell 2003). The authors concluded that dogs have shown and are therefore capable of experiencing similar jealousy-like emotions as human children (Volling et al. 2002; Hart 2016a). Although this study indicated emotional affects linked to aggression, it is less obvious that there is also evidence for jealousy in dogs. On the one hand, although aggression and jealousy are strongly related in humans, other emotions like sadness and fear are also involved or sometimes expressed instead. Moreover, jealousy in humans is a complex social emotion that can be displayed when an individual is threatened with losing something of personal value and involves affective, behavioral, and cognitive components (Pfeiffer and Wong 1989; Harris 2004; Sun et al. 2016; Zheng et al. 2019). It is unclear whether such a complex emotion is plausible in nonhuman animals. On the other hand, aggressive behaviors do not only arise from jealousy (Jiang et al. 2018). Furthermore, in behavioral studies of jealousy, dogs have not always shown aggressive behavior toward the other dog (Harris and Prouvost 2014), especially if the other dog was only a realistic-looking fake dog (Prato-Previde, Nicotra, Fusar Poli, et al. 2018a). Finally, it remains unclear whether the activity evoked in the amygdala was specific to the social identity of the caregiver or merely reflected the negative contrast of a dog witnessing another dog receiving reward (Jiang et al. 2018). Here, we explicitly avoided the use of food as this may have triggered food resource defense responses (“envy-like”) rather than “jealousy-like” responses triggered by competition for the attachment figure.

## Materials and Methods

### Subjects

All our subjects (*N* = 12) were private pet dogs (for details, see Supplementary Table 1) and we recruited them from human caregivers living in Vienna and nearby via the Clever Dog Lab website and database. The dogs were of different breeds (9 Border Collies, 2 mixbreeds, 1 Australian Shepherd), both sexes (7 females, 5 males), and their age ranged from four to eleven years (see Supplementary Table 1).

### Ethical Statement

All reported experimental procedures were reviewed and approved by the institutional ethics and animal welfare committee in accordance with the GSP guidelines and national legislation based on a pilot study at the University of Vienna (ETK-19/03/2016–2, ETK-06/06/2017). The dogs’ caregivers gave written consent to participate in the studies before conducting the tests.

**Figure 1:**
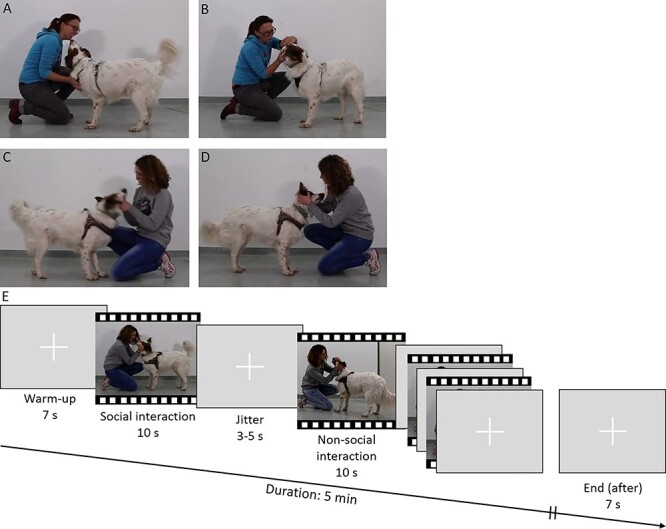
Human–dog interaction video scenes. Caregiver (*A* and *B*) and stranger (*C* and *D*) in the either social interaction (*A* and *C*) or nonsocial interaction (*B* and *D*); experimental design and time course of fMRI task (*E*).

### Stimuli

We recorded videos (duration = 10 s; 1280 × 720 pixels, 30 frames/s; Canon EOS 6D) of two different human–dog interactions under standardized conditions in the same room, at the same location in the room and constant lighting conditions. Thus, we controlled for visual, hue, satiation, or contrast differences in the videos. Furthermore, we used professional photo lamps to avoid visual distractions by shadows of the actors during the scenes (for more details see Supplementary Material, p. 2). During the experimental trials, the dogs watched these videos of their human caregiver (caregiver) or an unfamiliar person (stranger) interacting with a real dog in an either positive social (i.e., greeting and petting) or neutral nonsocial manner (i.e., a veterinary check, emotionlessly examining ears and teeth; see Fig. 1, Supplementary Material). The videos were shown in a randomized order and counter-balanced for the direction the human approached the dog (50% left, 50% right) and did not contain any sounds. The distance between the eyes of the tested dog and the screen was 85 cm and the visual stimuli were presented in the middle of the screen and the dogs’ view field (stimulus size: 1280 × 720 pixels) on a black background. The sex of the stranger (female) was matched to the sex of the majority of the human caregivers (9 females out of 10).

In an event-related fMRI design, the dogs viewed four different videos of interaction situations (duration = 10 s) depicting either the caregiver or a stranger in a social or nonsocial interaction. The task itself alternated between the four different interaction videos and a white fixation cross in the center of the screen on gray background, which served as a visual baseline (mean duration = 4 s, 3–5 s jitter). We divided the task into two experimental runs. If the dogs completed both runs within one session, a short break occurred between the runs. One run contained 20 trials (i.e., 5 per caregiver and stranger and 5 per social and non-social interaction). Each run ended with a visual baseline (duration = 7 s) and lasted approximately 5 min resulting in 300 whole-brain fMRI volumes. The order of the interaction video presentation was randomized. Note that one dog (Cliff) successfully completed only one of the two runs, and thus only the data from this run entered data analysis.

### fMRI Data Acquisition

MRI measurements were conducted in a 3 Tesla Siemens Skyra MR scanner (Siemens Healthineers) using the manufacturer’s 15-channel human knee coil. Functional volumes were acquired using a multiband echo planar imaging (EPI) sequence (multiband factor: 2) and obtained from 24 axial slices in descending order, covering the whole brain (interleaved acquisition) with a voxel size of 1.5 × 1.5 × 2 mm^3^ and a 20% slice gap (TR/TE = 1000 ms/38 ms, field of view = 144 × 144 × 58 mm^3^). We used an MR-compatible 32-inch screen (BOLDscreen 32 LCD, 120 Hz) at the head end of the scanner bore for the stimulus presentation. An eye-tracking camera (EyeLink 1000 Plus, SR Research) served to monitor the dog’s face/head and to control for movements and attentiveness during the scans. In case the tested dog was closing the eyes or intensely looking to the sides, top, or bottom of the screen, we immediately stopped and repeated the data collection (test run). Note that since the dogs were not trained to perform an eye-tracking calibration/validation procedure inside the MR scanner, we were not able to record the dogs’ eye gaze with the eye-tracker. The structural image of the individual dog’s brain was acquired in a prior scan session with a voxel size of 0.7 mm isotropic (TR/TE = 2100/3.13 ms, field of view = 230 × 230 × 165 mm^3^).

Before we conducted the fMRI task, the dogs went through an extensive training by a professional dog trainer to habituate them to the scanner environment and to stay motionless in the MR scanner for up to 8 min during data collection, as explained in detail in a previous publication (Karl et al. 2019). For data acquisition, unrestrained dogs laid down in prone position on the scanner bed with their head inside the human knee coil. During the entire procedure, the dogs were able to leave the scanner at any time by descending the custom-made ramp. The dog trainer stayed with the dog inside the scanner room throughout the entire testing process but outside of the dog’s visual field during the experimental runs. Data acquisition was immediately stopped and repeated when the dog moved extensively. Additionally, after each scan session, the realignment parameters were inspected. If we noticed that the overall movement exceeded the 3 mm threshold, the same test run was repeated in the next session. In the current study, all dogs completed their test runs (each 5 min) without any head movements exceeding 3 mm, and thus, the dogs’ brain activity of the entire test run(s) went into data analysis.

### fMRI Data Processing and Analysis

All imaging data were analyzed using *SPM12* (SPM, https://www.fil.ion.ucl.ac.uk/spm/software/spm12/) as described in Boch et al. (2021) and *MATLAB* 2014b (MathWorks). After slice-timing correction (referenced to the middle slice, Sladky et al. 2011) and image realignment, the functional images were manually reoriented to match the orientation of the canine breed-averaged template (Nitzsche et al. 2019) with the rostral commissure as a visual reference using SPM’s *Display* tool. We then manually skull-stripped the structural image using an individual binary brain mask for each dog, created with itk-SNAP (Yushkevich et al. 2006) with SPM’s *ImCalc* function. The structural image, the individual binary brain mask, and the functional imaging data were then coregistered to the mean image of each run. Next, the structural image was segmented (SPM12’s *Old Segmentation module*) into gray matter, white matter, and cerebrospinal fluid, using the tissue probability maps provided by the canine breed-averaged template (Nitzsche et al. 2019). We then normalized (SPM12’s *Old Segmentation module*) the functional and structural imaging data, along with the individual binary brain mask. Lastly, functional images were resliced (1.5 mm isotropic) and smoothed using a 3 mm Gaussian kernel (full-width-at-half-maximum, FWHM). Finally, we normalized the labels from another canine template (Czeibert et al. 2019) to the breed-average template space (Nitzsche et al. 2019), enabling a more detailed description of brain areas (see also Boch et al. 2021 for detailed description of the preprocessing workflow). The translation threshold (translational displacement along *x-*, *y-*, and *z*-axes) served as rough cutoff to decide if a run has to be repeated in a subsequent session. We did not specify a threshold for rotational displacement (pitch, raw, and roll). However, to additionally account for head motion, rotational displacements were converted from degrees to millimeters and we then calculated the scan-to-scan motion for each dog, referring to the frame-wise displacement (FD) between the current scan *t* and its preceding scan *t* − 1. Thus, frame-wise displacement accounts for both rotational and translational displacements.

**Figure 2:**
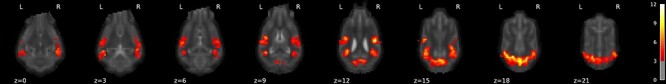
Viewing of human–dog interaction. Compared to baseline, activation was observed in the ectomarginalis, splenialis, sylvius, ectosylvius, and suprasylvius gyri (see Table 1). Statistical parametric maps were thresholded on voxel-level at *P* < 0.005 uncorrected and on cluster-level at *P* < 0.05 FWE-corrected for multiple comparisons.

Data analyses were performed in *Nipype* (Nipype, https://nipype.github.io; Gorgolewski et al. 2011) and *Nilearn* (Nilearn, https://nilearn.github.io) using the general linear model (GLM) approach implemented in SPM. The first-level, single-subject design matrix contained four task regressors: social caregiver, social stranger, nonsocial caregiver, nonsocial stranger. All trials were time-locked to the onset of each event, estimated as a boxcar function and convolved with a hemodynamic response function (HRF) specifically tailored for canine fMRI (Boch et al. 2021). As additional nuisance regressors, we included the six realignment parameters. We used normalized and individual binary brain masks (see above). SPM’s temporal high-pass filter with the default cutoff at 128 s was applied.

For group analyses, a series of *t*-tests based on single-subject contrasts were created to investigate the relevant effects of interest. These consisted of 1) one sample *t*-tests for the average activation of all conditions against baseline, contrast (caregiver|stranger & social|nonsocial) > implicit baseline; 2) a paired-sample *t*-test for higher average activation in the social compared to the nonsocial conditions, contrast (caregiver|stranger & social) > (caregiver|stranger & nonsocial), and 3) a paired-sample *t*-test for the interaction term of our two-by-two factorial design (caregiver social—caregiver nonsocial) > (stranger social—stranger nonsocial).

Whole-brain activation was tested for significance using FWE-corrected cluster-level inference with a threshold set to *P* < 0.05 at a cluster-forming threshold set to *P* < 0.005. In addition, we conducted a volume of interest (VOI) analysis based on the first-level parameter estimates in the following brain areas that have been implicated before as being relevant for affective responses and social cognition: amygdala, hippocampus, nucleus caudatus, insular cortex, and gyrus ectosylvius medius (Czeibert et al. 2019).

**Table 1:** Viewing of human–dog interaction

		Template space [mm]			
Cluster size [mm^3^]	Peak [*t*]	X	Y	Z	Structure
6682	12.88	−8.5	−28.5	+19.0	Gyrus ectomarginalis L
	11.46	+0.5	−31.5	+16.0	Gyrus splenialis R
	10.66	−17.5	−24.0	+16.0	Gyrus suprasylvius medius L
	10.63	+18.5	−9.0	+11.5	Gyrus ectosylvius rostralis R
999	8.12	−16.0	−7.5	+13.0	Gyrus ectosylvius rostralis L
	7.33	−20.5	−10.5	+7.0	Gyrus sylvius caudalis L

## Results

### Viewing of Human–Dog Interaction

To observe the BOLD response to the video stimulus compared with the fixation cross baseline, we created a contrast for the average effect over all four conditions, that is, (caregiver|stranger & social|nonsocial) > baseline. We observed significant activation in two bilateral clusters comprising the gyrus ectomarginalis, gyrus splenialis, and gyrus ectosylvius rostralis (see Fig. 2 and Table 1).

### Social versus Nonsocial Interactions

**Figure 3:**
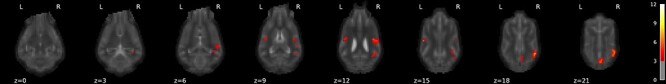
Social versus Nonsocial Interactions. Activation was observed in four bilateral clusters comprising the gyrus suprasylvius, gyrus sylvius rostralis, and gyrus ectosylvius rostralis (see Table 2). Statistical parametric map was thresholded on voxel-level at *P* < 0.005 uncorrected and on cluster-level at *P* < 0.05 FWE-corrected for multiple comparisons.

To observe the difference in BOLD response between a social versus nonsocial condition irrespective of the person shown, we calculated the contrast (caregiver|stranger & social) > (caregiver|stranger & nonsocial). We observed significant activation in four bilateral clusters comprising the gyrus suprasylvius, gyrus sylvius rostralis, and gyrus ectosylvius rostralis (see Fig. 3 and Table 2).

### Caregiver versus Stranger

To observe the difference in BOLD response between caregiver versus stranger irrespective of the interaction type, we calculated the contrast (caregiver & social|nonsocial) > (stranger & social|nonsocial). Using cluster-level correction, we did not observe any significant differences. With a more liberal threshold (*P* < 0.005, uncorrected), we observed a peak in both amygdalae (left: *T* = 4.25, right: *T* = 3.75) on the borderline to the lobus piriformis.

### Interaction: Caregiver (Social—Nonsocial) > Stranger (Social—Nonsocial)

**Figure 4:**
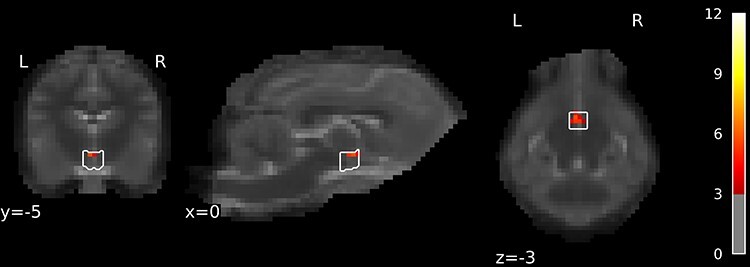
Interaction of (Caregiver vs. Stranger) versus (Social vs. Nonsocial). Contrast was defined as (Caregiver Social—Caregiver Nonsocial)—(Stranger Social—Stranger Nonsocial). Hypothalamus activation was found for the target contrast, *t* = 5.68, *P* = 0.00007, cluster size = 27 mm^3^ or 8 voxels, surviving *P* < 0.05 FWE small volume correction using the hypothalamus anatomical mask. Additionally, a VOI analysis for the hypothalamus revealed a significant effect for the target contrast *t* = 1.83, *P* < 0.0473.

To observe the difference in BOLD response between the caregiver and stranger for the social versus nonsocial contrast, we calculated the contrast (Caregiver Social—Caregiver Nonsocial) > (Stranger Social—Stranger Nonsocial). We observed significant activation in an anatomical area labeled as *diencephalon,* which however did not include the thalamus (as defined by the atlas used for determination of the anatomical structures). Due to the location of this active cluster ventral to the thalamus, we identified it as the BOLD response originating from neural activation in the hypothalamus (see Fig. 4 and Supplementary Figs 1 and 2, Uemura 2015).

### Volume of Interest Analyses

**Figure 5:**
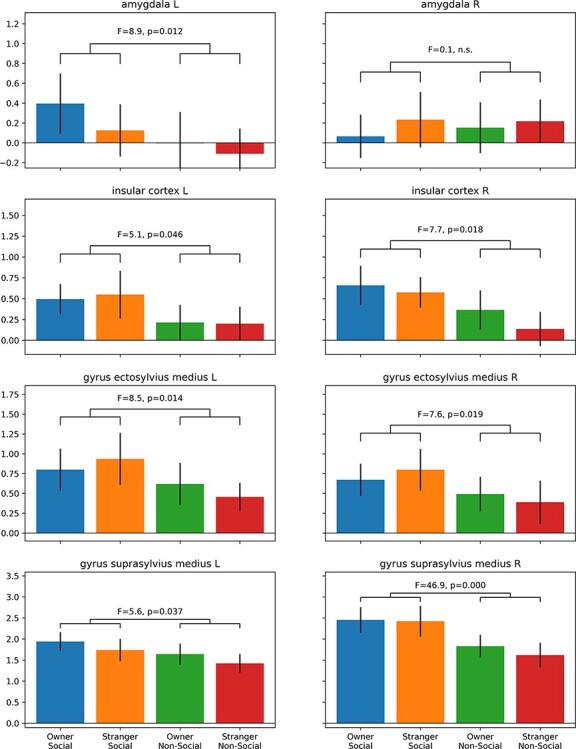
Volume of interest analysis. Activation for the social versus nonsocial conditions was increased in the left amygdala, bilateral insular cortex, bilateral ectosylvian gyrus and bilateral gyrus suprasylvian medius. *Y*-axes depict parameter estimates [a.u.] and error bars indicate the standard error of the mean.

Finally, based on anatomical masks from the atlas, we investigated the difference in smaller volumes of interests that have been implicated in affective responses and social cognition. Using a 2-by-2 repeated measures ANOVA, with factors social interaction and person, a significant main effect for the factor social interaction in the amygdala, insular cortex, gyrus ectosylvius medius, and gyrus suprasylvius medius was revealed (see Fig. 5; activation always higher during the social compared with the nonsocial condition). There were no significant effects in the hippocampus (left: *P* = 0.665, right: *P* = 0.758) and nucleus caudatus (left: *P* = 0.398, right: *P* = 0.823), and no main effect of the factor person, nor a significant interaction (all *P*-values > 0.05).

**Table 2:** Social versus nonsocial interactions

			Template space [mm]			
	Cluster size [mm^3^]	Peak [*t*]	X	Y	Z	Structure
1	442	7.22	+17.0	−24.0	+19.0	Gyrus suprasylvius medius R
		4.82	+14.0	−22.5	+4.0	
		4.66	+20.0	−15.0	+8.5	Gyrus sylvius rostralis R
		4.59	+17.0	−25.5	+11.5	
2	155	6.48	+2.0	−31.5	+19.0	
3	138	5.99	−19.0	−9.0	+14.5	Gyrus ectosylvius rostralis L
4	162	5.42	+15.5	−7.5	+11.5	Gyrus ectosylvius rostralis R

## Discussion

In this fMRI study, we explored the brain responses of pet dogs when witnessing their caregiver engage in an affectionate social interaction with another dog, assuming that this dog could be perceived as a potential rival and a threat to their social bond with the caregiver. As our main interest in this follow-up of a previous study about the neural underpinnings of dog–human attachment (Karl et al. 2020) was in the perception of the subjects’ caregiver and her/his behavior, we contrasted this with the same behaviors executed by a stranger. We hypothesized that witnessing the attachment figure enjoying a pleasant and socially engaging interaction with another dog would result in increased attention and arousal in comparison to the stranger executing the same behavior (to the same dog), as well as in comparison to the caregiver (and the stranger) showing a neutral nonsocial interaction simulating a veterinary check. This hypothesis rests on the assumption that the other dog is perceived as a social rival only in the first condition, and therefore the subject should show neural responses that can be associated with this perception and the putative risk of losing a valued relationship.

Overall, our analyses revealed brain responses that differed between the four different conditions. First and foremost, in comparison to the baseline, we found significant overall activation in two large bilateral clusters comprising the gyrus ectomarginalis, gyrus splenialis, gyrus sylvius, gyrus suprasylvius, and the gyrus ectosylvius rostralis. These activations indicate that the dogs’ brains are responsive to our visual scene presentation (e.g., Bunford et al. 2020). The fact that activation was found outside primary visual areas, which respond to low-level features of the stimulus material, indicates the engagement of regions required for the processing of more complex features. In fact, previous studies have already suggested that these areas could be associated with higher-order visual processing as well as with neocortical attention processes (e.g., Hecht et al. 2019; Karl et al. 2020; Boch et al. 2021). This provides the backbone of the more specific results, as it suggests that the dog brains overall responded to the shown scenes in ways that are indicative of processing of specific higher-level features. Comparing the social with the nonsocial conditions revealed activation in the gyrus suprasylvius, gyrus sylvius rostralis, and gyrus ectosylvius rostralis. The gyrus ectosylvius and the gyrus suprasylvius are brain regions of the lateral sensory cortex (located between the somatosensory, gustatory and auditory cortex) and are considered higher-order brain areas linked to perception and sensation (Hecht et al. 2019). Hecht et al. (2019) proposed that higher-order cortical areas like these might play a particular role in social interactions. In line with this interpretation and corroborated by our findings, other dog fMRI studies found multisensory activations in these brain areas during static or dynamic visual (i.e., human and dog faces) and auditory (i.e., con- or heterospecific vocalizations, nonvocal sounds) stimulus presentations (Andics et al. 2014, 2017; Cuaya et al. 2016, 2017; Thompkins et al. 2018; Bunford et al. 2020; Karl et al. 2020; Boch et al. 2021). Moreover, increasing the sensitivity of the analyses by a region of interest approach testing specific *a priori* hypotheses revealed additional differences between the social and nonsocial interaction in the amygdala, insular cortex, and gyrus ectosylvius, but not in the hippocampus or the nucleus caudatus. Note though that these differences were found irrespective of the human’s identity as caregiver versus stranger and thus were not based on an interaction effect. Such an effect was however revealed for the hypothalamus, where we found significantly increased activation for the caregiver versus the stranger for the social interaction contrasted with the nonsocial interaction. This finding is of particular relevance as it identifies the hypothalamus as a brain area that shows the strongest response in a situation where the caregiver interacts in a positive way with another dog (Karlsson et al. 2010)—and thus fits squarely with our hypothesis that the perceiver dogs may process this situation and the putative rivalry as particularly salient. Furthermore, this is in line with the results of our previous fMRI study in pet dogs showing that the human caregiver seems to play a superior role for the dogs (Karl et al. 2020). Finally, we found a tendency for a difference between the human identities in the amygdala, i.e., caregiver versus stranger, irrespective of the social situation. However, while this would be in line with previous research (Cook et al. 2018), this result was only detected at a liberal threshold and not corroborated by the ROI analysis, which is why we refrain from putting too much weight on it. Thus, although we cannot completely prove that the subject dogs recognized their caregivers in the displayed videos as in our previous study (Karl et al. 2020), there is already sufficient indirect evidence in the literature for pet dogs being able to recognize their caregiver’s face when compared to other human faces (Huber et al. 2013; Mongillo et al. 2017; Eatherington et al. 2020).

Overall, these findings provide evidence for our main hypotheses concerning an effect of the short videos showing different interactions between a human and a conspecific. Especially the type of interaction seemed to play a major role, since the dogs based on the increased activations in the neocortical as well as the limbic brain areas showed higher attention, arousal, and salience attribution toward the social positive than the nonsocial neutral human–dog interaction (Lamm and Singer 2010; Saper and Lowell 2014; Janak and Tye 2015).

However, the observed significant limbic brain area activations for the social versus nonsocial interactions are in line with more recent studies in humans and nonhuman animals showing that the amygdala along with the insular cortex is involved in salience attribution, and affective processing, and that this may channel into reward processing and learning about possible beneficial biological values and outcomes of stimuli (see reviews, Baxter and Murray 2002; Murray 2007; Salzman and Fusi 2010; Strigo and Craig 2016). More specifically, concerning the amygdala, the literature suggests this area to play an important role in affective processing and more generally the detection of and the physiological responses to salient stimuli (Sander et al. 2003; Lim et al. 2009). Human studies show that the amygdala enables an emotional evaluation but also memory consolidation of emotionally arousing events (Hamann et al. 1999; Anderson 2001; Karlsson et al. 2010), independent of the valence. Early amygdala lesion studies in humans and rodents also suggested a strong conservation of the function of the amygdala across vertebrate species, and this has, for example, been demonstrated for fear conditioning (reviewed in Janak and Tye 2015). Interestingly, in male monkeys, the amygdala, together with a few other brain areas such as the bilateral insula, is activated when they were confronted with threats to their exclusive sexual access to a female mate (Rilling et al. 2004). Our findings of the dogs’ increased amygdala activation could therefore also indicate a response to the seeing of another dog as a threat to their exclusive access to their human attachment figure and/or the high relevance of a situation where the caregiver interacts with another dog.

The insular cortex has been extensively linked to the processing and homeostatic regulation of affect and emotions, and in humans, it has even been suggested to play a key role in social affect and behavior, such as during empathy, cooperation, and affection (Lamm and Singer 2010). More specifically, this brain area seems to interoceptively track the bodily signals associated with emotions and affective states, as well as to trigger and guide homeostatic control to the affective states via regulation of the bodily responses (for review, see Strigo and Craig 2016). Pathological jealousy has been associated with altered dopaminergic frontostriatal reward circuitry and in the ventral medial prefrontal cortex (vmPFC) and insula involved in mentalizing/self-related processing and interoception/salience processing, respectively (Marazziti et al. 2013). Being confronted with social challenges such as social bonding and relationship maintenance involves the need for certain emotional responses that are intrinsically connected with sociality (DeSteno et al. 2006). The specific insular activations found in our dogs are therefore highly interesting with respect to these findings. They suggest that the social interaction led to a stronger call for homeostatic regulation. Note however that we cannot make any claims about the specific type of emotions, such as jealousy or envy, because of the absence of a statistical interaction effect (between social situation and human’s identity) for the insular activation.

This is why the differential engagement of the hypothalamus is of particular relevance. This subcortical brain area is linked to homeostatic regulation, which it exerts by means of autonomic nervous system and behavioral control (instantiated via specific neural circuits but also via neuroendocrine systems; Saper and Lowell 2014). In humans, greater activation of the hypothalamus has been found (in men more than in women) during the experience of jealousy (Takahashi et al. 2006). Hence, the highest activation of this area during the affectionate social interaction of the caregiver with the potential rival would be in line with an increased need for homeostatic regulation and the associated affective responses. This interpretation, however, needs further confirmation and linkage to measures of autonomic responses that we could not perform here due to the already demanding measurement setting of the fMRI scanner.

Social relationships provide many advantages in group-living nonhuman animals and humans, for example, increased protection, well-being, and health for the individual and therefore will be most likely protected and intended to maintain (Cohen 2004; Pusey 2005). In humans, Buck (1999) assumed that prosocial affects including, for example, attachment, represent the biological fundamentals of more complex social affect and emotions such as jealousy, envy, and guilt. These emotions are usually related to the feelings, thoughts, and actions of others (Hareli and Parkinson 2008). The specific activations in the amygdala, hypothalamus, and insular cortex are interesting with respect to the reported brain activations found in humans during the experience of jealousy (e.g., Takahashi et al. 2006; Zheng et al. 2019).

Several studies in human infants from the age of six to twelve months showed that when the mother directed her attention to a realistic-looking doll as a social test partner, infants aimed for closer proximity to their mother, including increased approach, touch, and gaze and showed higher reactivity levels, for example, arousal, aggression, and more negative emotions (anger, sadness) and affect compared to a nonsocial object such as a book (Hart et al. 1998, 2004; Hart 2016a; Mize and Jones 2012; Mize et al. 2014). Furthermore, their stress-induced facial expressions and vocalizations indicated a decrease in joy. Still, when the mother was holding a book or a stranger directed the attention to the doll, infants showed the same responses but at a lower level (Hart et al. 1998; Hart 2010).

The findings of several behavioral studies suggested that the human–dog relationship resembles the human mother–child bond by forming a stable attachment bond to their caregivers (e.g., Bowlby 1958; Topál et al. 1998; Gácsi et al. 2001; Prato-Previde et al. 2003; Palestrini et al. 2005; Palmer and Custance 2008; Horn et al. 2013). Based on this strong human–dog relationship, we may conclude that pet dogs also try to protect and maintain this meaningful relationship and therefore would negatively react to a potential threat of weakening it or at least of losing the exclusive affective attention of the caregiver. Harris and Prouvost (2014) proposed that jealousy-related behaviors either developed to protect pair-bonded sexual relationships from potential rivals or jealousy evolved in species with dependent offspring that competes for parental resources, for example, attention, care, food, or which form alliances and perform cooperation with other group members to survive.

In conclusion, the lack of widely agreed criteria for assessing nonhuman emotions, especially complex ones, affords a very careful and parsimonious interpretation of our findings. Even the terminology for human higher-order emotions is not always consistent (Hareli and Parkinson 2008; Anderson and Adolphs 2014; Watson and Stanton 2017; Sznycer 2019), but describing and investigating animal emotions is even more challenging since nonhuman animals cannot tell whether or what kind of emotions they experience (Paul and Mendl 2018). In contrast to human emotion research, animal researchers face a lack of subjective self-reports about emotional experiences, distinct emotional indicators, or comparable species-specific emotional facial expressions. Studies showed that there seem to be similarities in facial expressions in closely related primate species but not across mammals (*cats*: Caeiro, Burrows, et al. 2017b; *dogs*: Caeiro, Guo, et al. 2017a; *rhesus macaques*: Parr et al. 2010; *chimpanzees*: Parr et al. 2007). In particular, there is no specific jealousy-related facial expression or any other specific jealousy defining criterion to reliably identify or measure it. Therefore, reliably detecting and measuring such an emotion in dogs remains difficult and highly speculative. To date, we do not have any gold standard method to measure emotions and ideally one would need to assess all internal (e.g., neural and physiological responses) and external (e.g., behavioral expressions) changes and processes while experiencing an emotion (reviewed by Scherer 2005).

Therefore, the minimum conclusion from our study is that dogs not only seem to be sensitive to the difference between a social and nonsocial behavior of a human toward a conspecific, but that they also showed condition-specific arousal and probably were emotionally affected by the different scenes. Note, due to head size restriction according to the coil dimension, our dog sample mainly consists of herding dogs, that is, Border Collies, Australian Shepherd, and therefore we cannot know if our findings are specific for this kind of dog breeds. Thus, future tests with various other dog breeds are needed to be able to generalize our results to other pet dogs.

In summary, and although preliminary, our findings provide a first view into the neural correlates of social and emotional processing in dogs and their capabilities to distinguish between different affective human–dog interactions. Additional studies are needed to further investigate dogs’ emotional responses to social stimuli and the cognitive abilities to recognize and identify specific individuals, for example, the dog’s caregiver, shown in complex interactions or situations.

## Supplementary Material

210628_Supplemental_Material_Cerebral_Cortex_Comm_tgab047

Supplementary_movie_S1_social_tgab047

Supplementary_movie_S2_non_social_tgab047

## Data Availability

Supplementary details are included in the Supplementary Material files of this article (see Supplementary Tables 1–5, Supplementary Figs 1 and 2, and Supplementary Movies 1 and 2). Additionally, unthresholded statistical maps have been uploaded to OSF.io and are available at osf.io/jwm36/.

## Notes

S.K., C.L., and L.H. designed the research; S.K. collected the data; R.S. analyzed the data; S.K., R.S., L.H., and C.L. drafted the manuscript; all authors discussed the results and contributed to the final manuscript. Conflict of Interest: The authors declare no competing interests.

We thank Velia Nicotra and Paola Valsecchi for earlier work on the video stimuli and their design, Karin Bayer for administrative support and Wolfgang Berger and Peter Füreder for technical help, Alexa Huber-Müllner and Ekaterina Pronizius for stimuli and experimental design support, Magdalena Boch, Morris Krainz, and several student apprentices for help with the fMRI data collection. Additionally, we sincerely thank the dogs’ caregivers and their dogs for participating in our study. *Conflict of Interest*: The authors declare no conflict of interest.
