# Supplementary material for: Neural Responses of Pet Dogs Witnessing Their Caregiver’s Positive Interactions with a Conspecific: An fMRI Study
Source: Cereb Cortex Commun. 2021 Jul 19;2(3):tgab047. doi: 10.1093/texcom/tgab047 (PMC8382916; doi:10.1093/texcom/tgab047)
Supplement: 210628_Supplemental_Material_Cerebral_Cortex_Comm_tgab047 [file 210628_supplemental_material_cerebral_cortex_comm_tgab047.docx]

**Neural responses of pet dogs witnessing their caregiver’s positive interactions with a conspecific: an fMRI study**

Sabrina Karl, Ronald Sladky, Claus Lamm, and Ludwig Huber

**SUPPLEMENTARY MATERIAL**

**METHODS**

**Subjects**

The sample of subjects consisted of 12 pet dogs (see Table S1).

**Table S1.** Characteristics and experimental assignment of subjects.

| no. | dog | breed | sex | age when adopted  (in weeks) | neutered (yes/no) | age when tested in 2019  (in years) | info |  |
| --- | --- | --- | --- | --- | --- | --- | --- | --- |
| 1 | Aeden | Border Collie | m | 13 | yes | 11 |  |  |
| 2 | Amy8 | Border Collie | f | 8 | no | 9 |  |  |
| 3 | Cameron | Border Collie | m | 9.5 | no | 7 |  |  |
| 4 | Carlisle | Border Collie | m | 9 | no | 8 |  |  |
| 5 | Chasie | Border Collie | f | 12 | yes | 10 |  |  |
| 6 | Cliff | Border Collie | m | 8 | yes | 5 | Only one test run |  |
| 7 | Emily | Border Collie | f | 8 | yes | 11 |  |  |
| 8 | Kayleigh | Border Collie | f | 9 | yes | 8 |  |  |
| 9 | Kiki | mixbreed | f | 96 | no | 4 |  |  |
| 10 | Linus | Australian Shepherd | m | 10 | no | 5 |  |  |
| 11 | Maeva | mixbreed | f | 9 | yes | 8 |  |  |
| 12 | Ziva | Border Collie | f | 9 | yes | 8 |  |  |

**Stimuli video recording**

The experimenter (stranger) videos were recorded first and then shown to the dogs’ caregivers to copy the respective interaction style with the actor dog. Accordingly, we gave them specific instructions how to behave before the actual recording of the videos occurred. During the recording, we counted each second loudly so that the length of standing (at the beginning), walking and the interaction time was always the same and the caregivers knew how and when (at certain loudly counted numbers) to behave and interact with the dog. While recording, we tried to make sure that the caregivers acted as instructed and that both the human and the dog were always positioned straight to each other to be properly seen from the camera angle (later perceiver dogs’ perspective). If that was not the case, we immediately stopped the recording and repeated it. The camera was attached to a tripod and placed at the height of 80 cm to present the entire scene from an approximate subject dogs’ perspective/ head level to them. The actor dog presented in the videos was always the same dog and the experimenter was briefly familiar with him. The caregivers and the subject dogs were not familiar with the actor dog. During the recordings, the actor dog was trained to sit still until the human called him for the interaction which happened always at the same time of the recording and at the same location (confirmed with ground markings). We tried to make sure that all humans acted the same way (depending on interaction) and thus, the dog respectively behaved the same way with all humans as well.

**Determining the location of the hypothalamus**


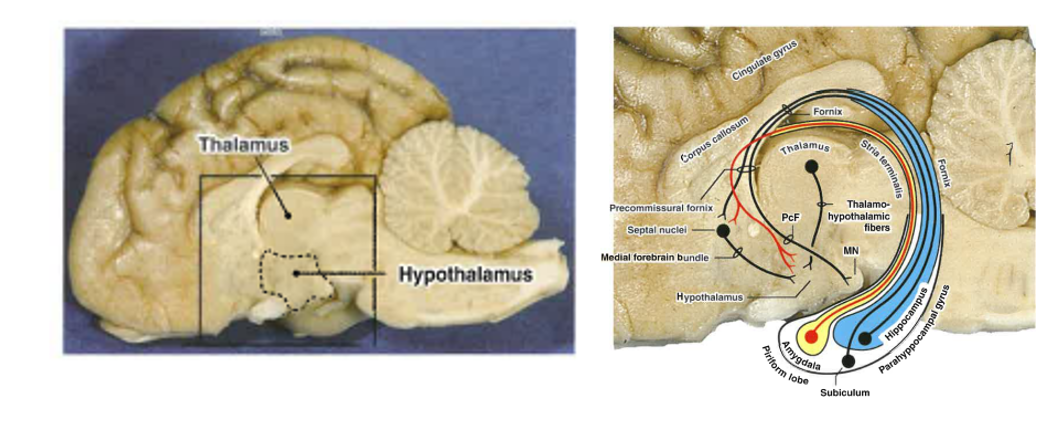


***Figure S1. Location and detailed structure of the hypothalamus in the dog’s brain (Uemura 2015, p. 373, 377)***

By explaining our analysis as a process of elimination we wanted to ensure that we are confident that our finding in the hypothalamus is not part of a super-threshold cluster in the thalamus that bleeds into the adjacent atlas region due to smoothing or improper alignment. To better substantiate our finding, we created an anatomical mask of the hypothalamus. The hypothalamus (yellow outline) was defined as a subregion of the diencephalon mask (red outline; see Figure S2) using Uemura 2015 (see Figure S1) as a guideline. The activation cluster remains within the hypothalamus (or diencephalon) mask and does not extend to the thalamus (blue outline; see Figure S2).


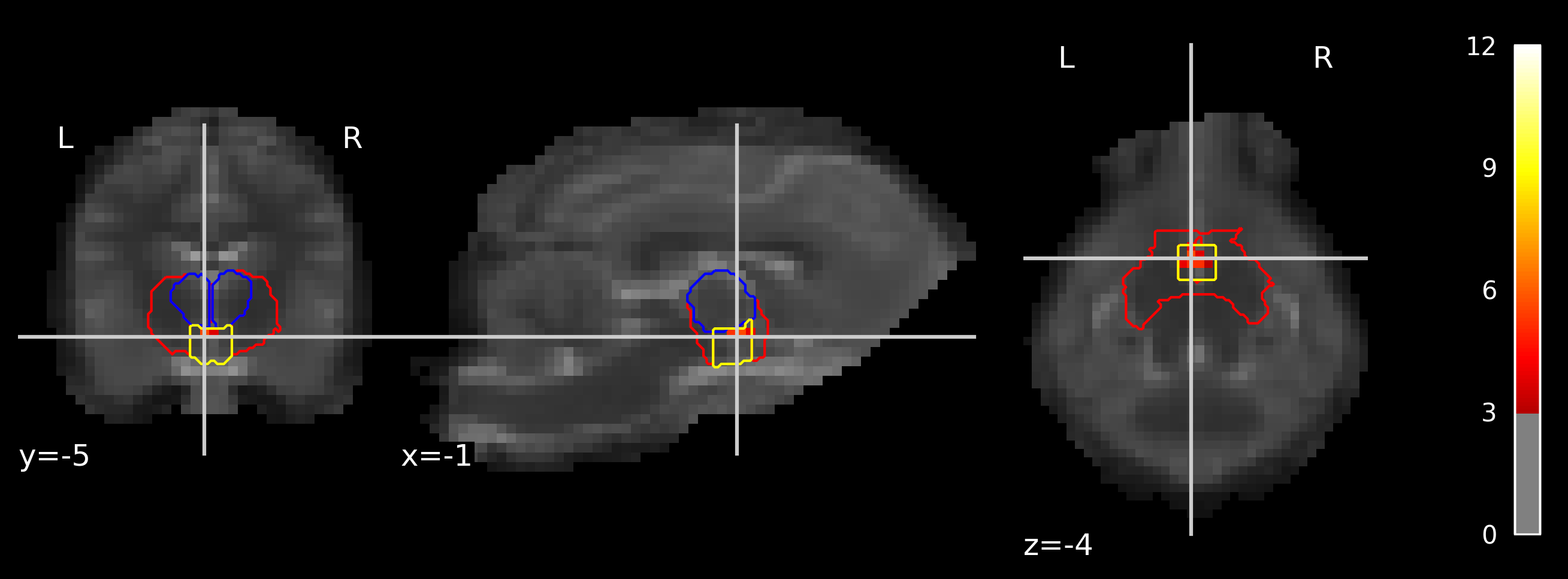


***Figure S2. Anatomical mask of the hypothalamus. Hypothalamus (yellow outline) as a subregion of the diencephalon mask (red outline) and the thalamus (blue outline) using Uemura 2015 as guideline.***

**Motion analysis of stimuli videos**

To rule out that the brain response of the perceiver dogs during the stimuli presentation was merely driven by any motion differences between the videos of the respective human-dog interaction type, we conducted two different methodological approaches and analyses to determine the level of motion in the videos.

First, we calculated the average standard deviation (STD) of the time of the motions of each video. Therefore, we extracted a time course for each pixel in the movie and calculated the standard deviation for each pixel. Then, we averaged over all standard deviations to quantify the average intensity changes over time. Videos with high motion have higher intensity variance than low motion videos. This allowed us to objectively compare and rule out motion differences between the stimulus conditions. Note, that during video recording the camera was attached to a tripod, thus, all movements seen in the videos were performed by the actors. According to our analysis, we could confirm that there was no effect of (difference between) the non-social and the social interactions (see Fig. S2a). Additionally, we performed a two sample t-test and found the same result (social vs. non-social: p=0.541). Consequently, the low level visual features of the movements were consistent between the displayed interactions in the videos.

***
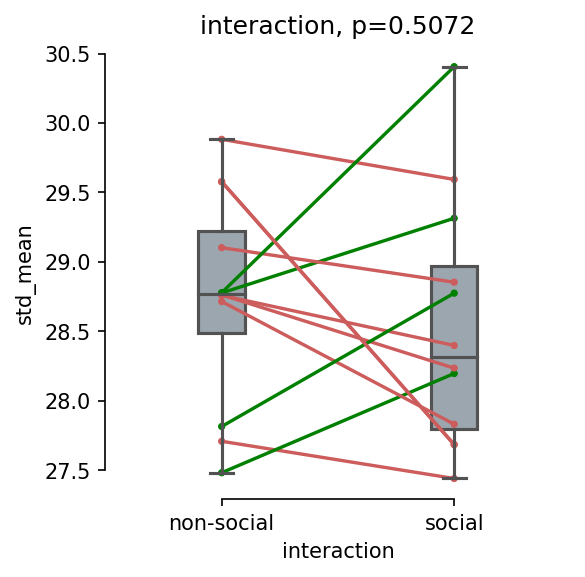
***

***Figure S2a. Statistical video motion comparison; average STD of motion time of the non-social vs. social interaction.***

Moreover, we compared the trajectories/ paths of the human movements during the two interaction styles with a motion analysis software ([www.kinovea.org](http://www.kinovea.org)). We marked and tracked the face/ head position (start point) of the human (caregiver and stranger) while she/ he moved during the interaction (end point) with the actor dog (see red line in Fig. S2b, c). The red line depicted down to the middle between the human and the dog represents the locomotion towards the interaction spot between human and dog and the diffuse end of the line represents the human’s motion during the two different interaction styles. This showed that the ends of the trajectory lines of both humans show more movement/ tracking (diffuse line endings, see Fig. S2b, c) during the social interaction than during the neutral interaction. These slightly increased, more active movements during the positive socially engaging interactions were intended as contrast/ comparison to the neutral, vetcheck interaction. They were necessary to realistically and naturally visualize the two different types of interactions to investigate whether the perceiver dogs recognize or discriminate between the different contexts (social vs. non-social). Besides, we showed that the movements towards the dog of each human were very similar despite different following interactions (similar red curves per person in Fig. S2b, c).

**
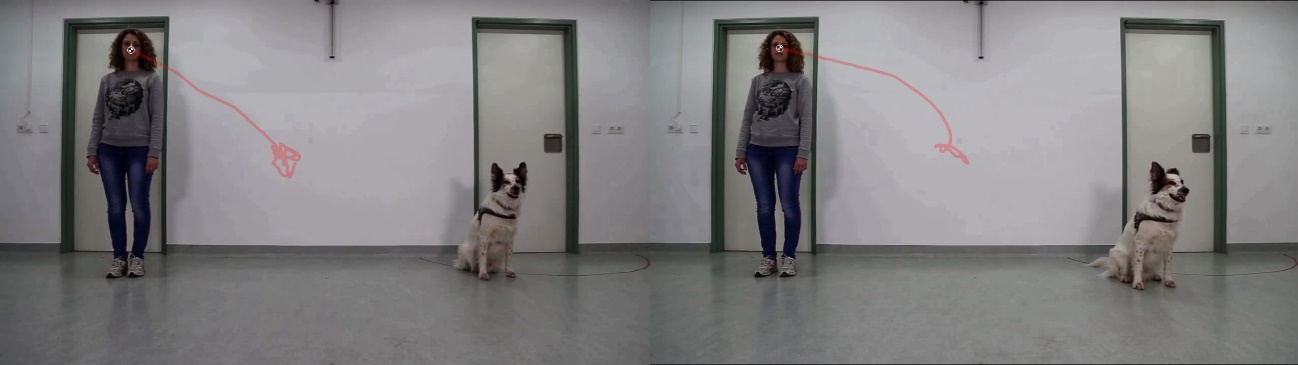
**

***Figure S2b. Trajectories (red line, start: face, end: interaction location) of the human’s (stranger) head motion during the two interaction styles, positive (left) vs. neutral (right). The shape of the end of the line between the human and the dog indicates the amount of movement during the interaction.***

**
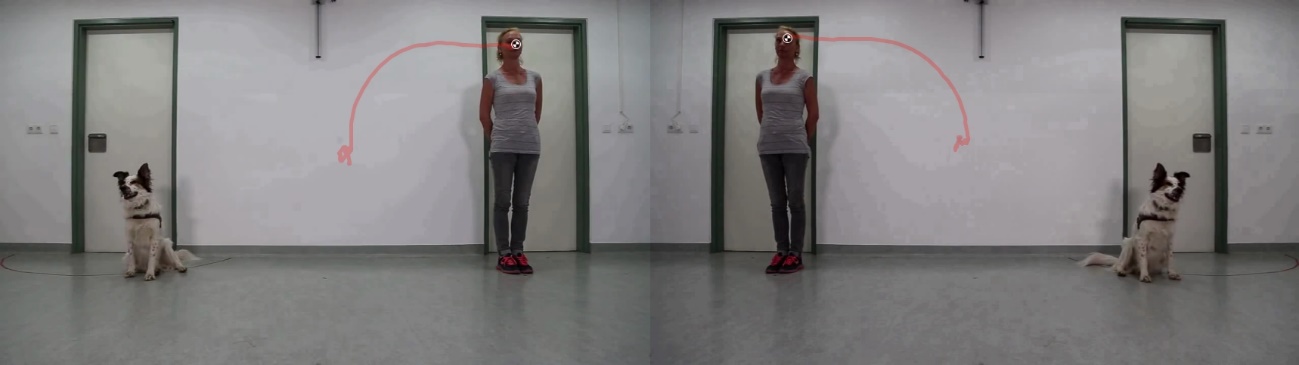
**

***Figure S2c. Trajectories (red line, start: face, end: interaction location) of the human’s (caregiver example) head motion during the two interaction styles, positive (left) vs. neutral (right). The shape of the end of the line between the human and the dog indicates the amount of movement during the interaction.***

**RESULTS**

**Table S2.** Caregiver Social Interaction.

|  | |  |  | **Template Space [mm]** | | |  |
| --- | --- | --- | --- | --- | --- | --- | --- |
|  | | **Cluster Size [mm^3^]** | **Peak [t]** | **X** | **Y** | **Z** | **Atlas** |
| 1 | | 6372 | 11.46 | +18.5 | -9.0 | +11.5 | gyrus ectosylvius rostralis R |
|  |  | | 11.26 | -8.5 | -28.5 | +19.0 | gyrus ectomarginalis L |
|  |  |  | 9.85 | +17.0 | -24.0 | +19.0 | gyrus suprasylvius medius R |
|  |  |  | 9.78 | +0.5 | -31.5 | +17.5 |  |
| 2 | | 1005 | 9.78 | -16.0 | -7.5 | +13.0 | gyrus ectosylvius rostralis L |
|  |  | | 8.27 | -20.5 | -10.5 | +7.0 | gyrus sylvius caudalis L |

**Table S3.** Caregiver Non-Social Interaction.

|  | |  |  | **Template Space [mm]** | | |  |
| --- | --- | --- | --- | --- | --- | --- | --- |
|  | | **Cluster Size [mm^3^]** | **Peak [t]** | **X** | **Y** | **Z** | **Atlas** |
| 1 | | 5275 | 13.80 | +0.5 | -31.5 | +16.0 | gyrus splenialis R |
|  |  | | 10.34 | +23.0 | -21.0 | -3.5 | gyrus suprasylvius caudalis R |
|  |  |  | 9.67 | -17.5 | -24.0 | +16.0 | gyrus suprasylvius medius L |
|  |  |  | 9.63 | -8.5 | -28.5 | +19.0 | gyrus ectomarginalis L |
| 2 | | 715 | 6.34 | -20.5 | -12.0 | +5.5 | gyrus sylvius caudalis L |
|  |  | | 5.93 | -16.0 | -7.5 | +11.5 | gyrus ectosylvius rostralis L |
|  |  |  | 4.89 | -17.5 | -7.5 | +2.5 | gyrus sylvius rostralis L |
|  |  |  | 4.09 | -22.0 | -18.0 | +2.5 | gyrus ectosylvius caudalis L |

**Table S4.** Stranger Social Interaction.

|  |  |  | **Template Space [mm]** | | |  |
| --- | --- | --- | --- | --- | --- | --- |
|  | **Cluster Size [mm^3^]** | **Peak [t]** | **X** | **Y** | **Z** | **Atlas** |
| 1 | 6051 | 11.23 | -13.0 | -19.5 | +13.0 | gyrus suprasylvius medius L |
|  |  | 10.98 | -8.5 | -28.5 | +20.5 | gyrus ectomarginalis L |
|  |  | 10.97 | +0.5 | -34.5 | +17.5 |  |
|  |  | 10.38 | +8.0 | -30.0 | +17.5 | gyrus ectomarginalis R |
| 2 | 847 | 9.06 | -16.0 | -4.5 | +8.5 | gyrus sylvius rostralis L |
|  |  | 6.26 | -22.0 | -15.0 | +5.5 |  |
|  |  | 4.35 | -25.0 | -9.0 | +13.0 |  |
|  |  | 3.95 | -19.0 | -6.0 | +4.0 | gyrus sylvius rostralis L |

**Table S5.** Stranger Non-Social Interaction.

|  | |  |  | **Template Space [mm]** | | |  |
| --- | --- | --- | --- | --- | --- | --- | --- |
|  | | **Cluster Size [mm^3^]** | **Peak [t]** | **X** | **Y** | **Z** | **Atlas** |
| 1 | | 4758 | 12.52 | -8.5 | -28.5 | +19.0 | gyrus ectomarginalis L |
|  |  | | 10.55 | +18.5 | -9.0 | +11.5 | gyrus ectosylvius rostralis R |
|  |  |  | 9.75 | -14.5 | -22.5 | +11.5 | gyrus suprasylvius medius L |
|  |  |  | 9.13 | +0.5 | -31.5 | +16.0 | gyrus splenialis R |
| 2 | | 600 | 9.00 | -20.5 | -12.0 | +5.5 | gyrus sylvius caudalis L |
|  |  | | 6.78 | -17.5 | -6.0 | +10.0 |  |

**Supplementary Movie Captions**

**Movie S1.** Video showing a positive social human-dog interaction.

**Movie S2.** Video showing a neutral non-social human-dog interaction.

**References**

Uemura EE. 2015. Fundamentals of Canine Neuroanatomy and Neurophysiology. Ames, Iowa: John Wiley & Sons, Inc.
